# A nomogram for predicting screw loosening after single-level posterior lumbar interbody fusion utilizing cortical bone trajectory screw: A minimum 2-year follow-up study

**DOI:** 10.3389/fsurg.2022.950129

**Published:** 2022-09-13

**Authors:** Yiqi Zhang, Yue Li, Yong Hai, Li Guan, Xinuo Zhang, Aixing Pan, Hongyi Lu, Bingchao Wu, Yuzeng Liu

**Affiliations:** ^1^ Department of Orthopedics, Beijing Chaoyang Hospital, Capital Medical University, Beijing, China; ^2^ Department of Orthopedics, The General Hospital of Taiyuan Iron / Steel (Group) Corporation, Taiyuan, China

**Keywords:** cortical bone trajectory, screw loosening, lumbar spine, nomogram, spine

## Abstract

**Purpose:**

This study aims to investigate the risk factors for screw loosening after single-level posterior lumbar interbody fusion (PLIF) utilizing cortical bone trajectory (CBT) screw and establish a nomogram for predicting screw loosening.

**Methods:**

A total of 79 patients (316 screws) who underwent single-level PLIF with CBT screw were included in the study. Preoperative, postoperative, and final follow-up demographic data, surgical data, and radiographic parameters were documented and analyzed to identify risk factors, and a predictive nomogram was established for screw loosening. The nomogram was assessed by concordance index (C-index), calibration plot, decision curve analysis (DCA), and internal validation.

**Results:**

The incidence of screw loosening was 26.6% in 79 patients and 11.4% in 316 screws. Multifactorial regression analysis confirmed that fixed to S1 (FS1, OR = 3.82, 95% CI 1.12–12.71, *P* = 0.029), the coronal angle of the screw (CA, OR = 1.07, 95% CI 1.01–1.14, *P* = 0.039), and cortical bone contacted layers (CBCLs, OR = 0.17, 95% CI 0.10–0.29, *P* < 0.001) were risk factors and incorporated in the nomogram for predicting screw loosening after single-level PLIF with a CBT screw. The C-index of the nomogram was 0.877 (95% CI 0.818–0.936), which demonstrated good predictive accuracy. The calibration plot indicated an acceptable calibration of the nomogram that also had a positive benefit in guiding treatment decisions.

**Conclusion:**

FS1, CA, and CBCLs are identified to be significant risk factors for screw loosening after single-level PLIF with the CBT technique. The nomogram we have established can be used to predict screw loosening and contribute to surgical decisions.

## Introduction

Cortical bone trajectory (CBT) is an alternative approach first proposed by Santoni et al. as the treatment for a lumbar degenerative disease (1). CBT screw was inserted *via* the trajectory that could engage the pars, medial, and superior cortices of the pedicle isthmus for spinal fusion, and theoretically, it provided comparable pull-out resistance and stability to a traditional pedicle screw (TPS) (2–4). Likewise, good results have been found in literature works reporting the application of CBT screw in osteoporosis lumbar spine (1, 5, 6). The main role of the screw in lumbar fusion is to reduce the motion of the spine and to conduct the stabilization, whereas screw loosening is observed in quite a few literature works (7–9). As reported, the incidence of screw loosening in TPS was 1%–60% (7, 10, 11), and risk factors were related to osteoporosis, sacrum instrument, excessive load, and local high strains; however, it was not unified. CBT screw conducts a comparable fixation to TPS according to the characteristic, and it may reduce the risk of screw loosening due to the loading resistivity of cortical bone of the pedicles. Nevertheless, the screw loosening rate was still observed to be 62.5% (9, 12–14), and the risk factors were also uncertain. This leads to consideration of the differences in risk factors for screw loosening between CBT screw and TPS.

Screw loosening in both the TPS and CBT screw may require revision surgery due to symptomatic spinal instability and instrument failure (9, 11, 15, 16); thus, comprehension of screw loosening is essential. Previous studies were researched, and we found that few studies have concentrated on CBT screw loosening, or most of them lacked long-term follow-up and sufficient evidence. Hence, clear exploration of screw loosening of CBT screws is demanded with an available analysis of the literature.

The present study aimed to detect the prevalence of screw loosening in single-level PLIF using CBT screw with a minimum of 2 years of follow-up and to establish a nomogram for predicting screw loosening individually in each vertebra.

## Methods

**Figure 1:**
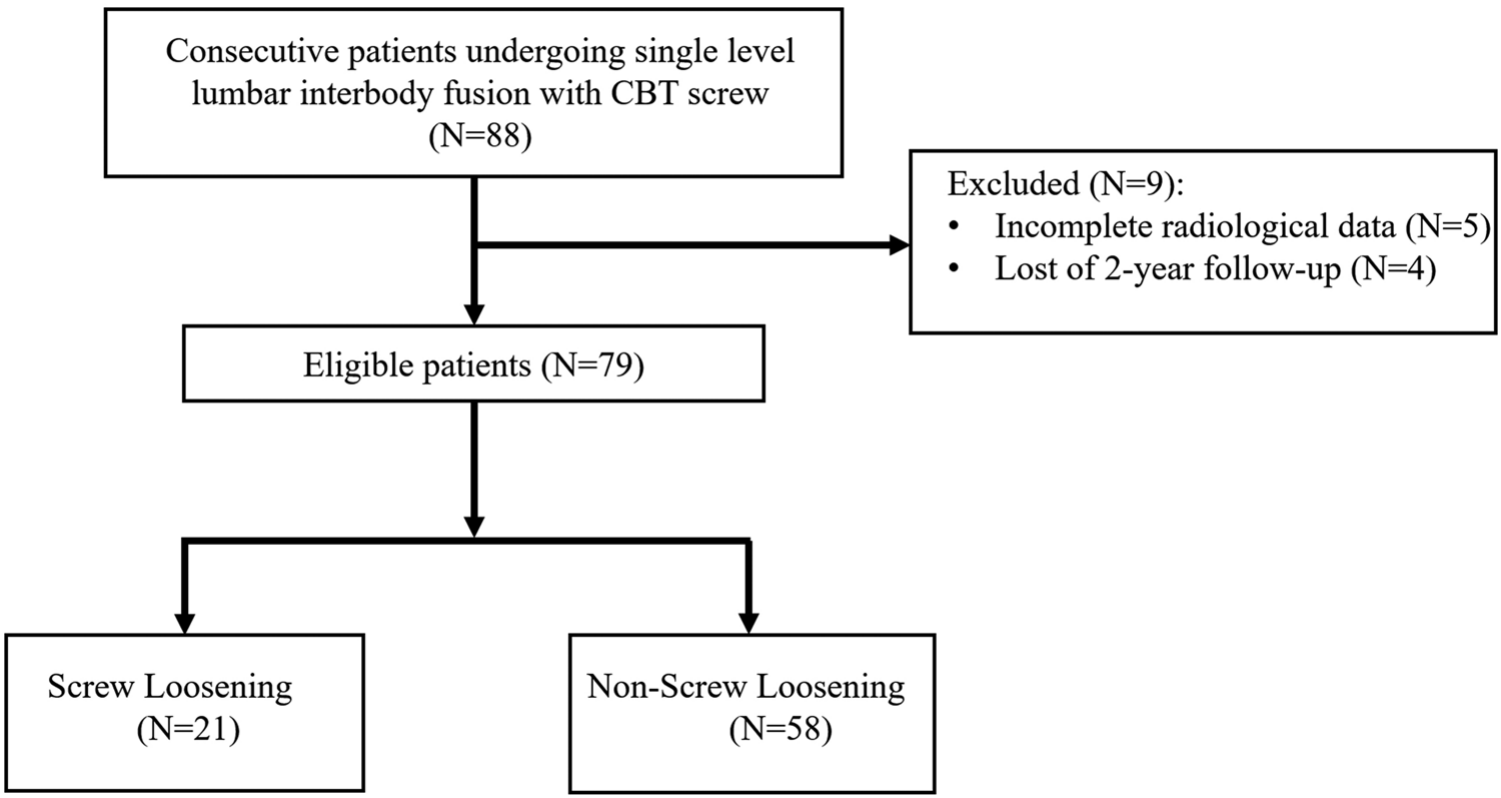
Flowchart of the study.

This was a retrospective study in the institution. A total of 88 consecutive patients were included in the study from November 2017 to January 2020, and 79 eligible patients were evaluated (Figure 1). Inclusion criteria are listed as follows: (1) patients diagnosed with lumbar degenerative disease (lumbar disc herniation, lumbar spinal stenosis, and lumbar spondylolisthesis) and who underwent single-level PLIF with CBT screws; (2) minimum follow-up time of above 2 years. Excluded criteria are as follows: (1) incomplete radiological data; (2) patients who underwent surgery diagnosed with lumbar infection, lumbar vertebral tumor, or history of lumbar surgery. The study was approved by the institutional review board of the hospital.

### Surgical technique

The patient was placed in a prone position. A 5-cm midline skin incision was performed in the lumbar area. Muscular dissection was performed until the vertebral isthmus was exposed. The facet joints were exposed, and adjacent facet joints of the fusion area were avoided. The entry point was selected as an intersection of a vertical line through the center of the inferior facet joint of the adjacent cephalic vertebra and a horizontal line 3–4 mm below the inferior facet joint of the cephalic vertebra (a notch might be identified on the isthmus). The track was drilled with a 2 mm burr into the cortical bone with an approximate 10°–15° angle from medial to lateral and 20°–25° angle from caudal to cranial. Locating pins were placed into the track, and fluoroscopy was performed to check the position. Then, decompression was performed, and a cage (PEEK) filled with autogenous bone was implanted into the intervertebral space after endplate preparation and autogenous bone insertion. After the decompression, pins were removed, and CBT screws (for the S1 vertebra, the screw was 45 mm in length and 6.0 mm in diameter, and for other vertebras, the screw was 35 mm in length and 5.5 mm in diameter) were inserted through the tracks with spinous process preservation. Bended rods were then positioned and tightened bilaterally after compression was performed. Finally, fluoroscopy was performed to recheck the position of the screws and cage before the skin was sutured layer by layer.

### Clinical and radiological evaluations

**Figure 2:**
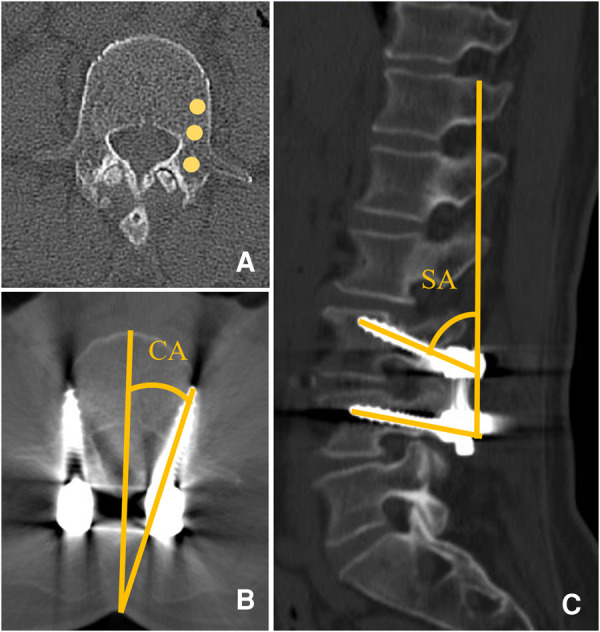
(**A**) HU measurement of an average of three points identified according to the screw track in the preoperative lumbar CT scan. (**B**) CA was defined as the angle between the screw and spinous process in the axial plane. (**C**) SA was defined as the angle between the screw line and vertical line in the sagittal plane.

**Figure 3:**
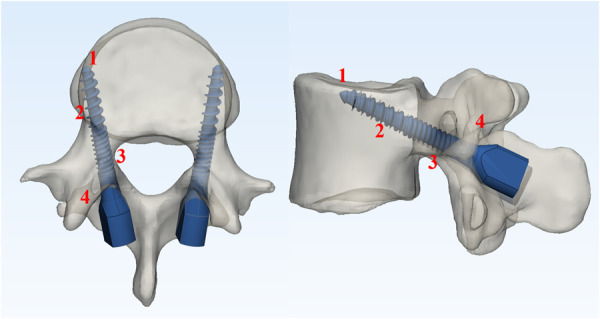
3D illustration of the maximum contact of cortical bone.

Clinical, demographic, and surgical data including age, gender, body mass index (BMI), operation time, and estimated blood loss (EBL) were collected. Radiological parameters including the coronal angle of the screw (CA), sagittal angle of the screw (SA), fixed to S1 (FS1), Hounsfield unit (HU) measurement of the trabecular bone of screw location, and cortical bone contacted layers (CBCLs) were evaluated (Figures 2, 3). The HU measurement was defined as the average of three points located in the screw track in a preoperative CT scan. Screw loosening was defined as a continuous lucent zone with a size of more than 1 mm and surrounded by a thin sclerotic zone in a CT scan (7, 17, 18). Bone fusion was graded according to Bridwell classification into three grades based on a lumbar CT scan (19): Grade I, complete fusion with the bridging bone bonding with both adjacent vertebral bodies; Grade II, incomplete fusion with the bridging bone bonding with either superior or inferior vertebral bodies; Grade III, failed fusion with incomplete bony bridging. Bone fusion was assessed by CT scan slices selected from the center of the cage or the largest bone grafting (20). The Oswestry Disability Index (ODI) was used to evaluate back pain preoperatively and at a postoperative time point of 6 months and final follow-up.

### Statistics analysis

SPSS Statistics Version 23.0 (IBM, Armonk, New York) and R software (version 4.1.2) were used for data analysis. Univariate analysis was performed with an independent *t*-test and Mann–Whitney *U* test for continuous data and discontinuous data, respectively, and quantitative data were listed as means ± SD with normal distribution or as medians with interquartile ranges with non-normal distribution. The chi-squared test was used for categorical data analysis.

Multivariate logistic analysis was further performed on variates that had significant differences in univariate analysis (*P* < 0.05). In this study, CBCLs, CA, and FS1 were put into the logistic model. A multiple logistic regression model was applied to select significant variables with a stepwise forward method, and the odds ratio (OR) and 95% confidence interval (CI) of the variables were recorded. A nomogram was established with R software. The concordance index (C-index) of the nomogram was calculated to evaluate the predictive accuracy of the nomogram utilizing the “rms” package. A calibration plot and the Hosmer–Lemeshow test were used to assess the calibration, and the nomogram was internally validated by the bootstraps of 1,000 resamples. Decision curve analysis (DCA) was calculated by the “rmda” package to evaluate the clinical usefulness of the nomogram. The reliable outcome is considered as C-index >0.75, and *P* value <0.05 was considered statistically significant for all data.

## Results

A total of 88 consecutive patients were identified, and 79 patients (316 screws) were included in the study. The cohort contained 35 (44.3%) male patients with an average age of 65.14 ± 9.74 years, and the average BMI was 26.83 ± 4.49. The mean follow-up time was 25.38 ± 1.77 months (Table 1). The incidence of screw loosening was 26.6% (21) in the cohort and 11.4% (36) in 316 screws; 5 patients presented back pain and received conservative treatment, and the other patients (16) were asymptomatic. The patients were divided into two groups according to the presence of screw loosening: screw loosening group (SL) and no screw loosening group (non-SL). Statistically significant differences were found in CA (*P* = 0.039), FS1 (*P* = 0.029), and CBCLs (*P* < 0.001) between the two groups (Table 2). Multiple logistic regression was performed on these parameters, and the results demonstrated that FS1 (OR = 3.82, 95% CI 1.12–12.71, *P* = 0.029), CA (OR = 1.07, 95% CI 1.01–1.14, *P* = 0.039), and CBCLs (OR = 0.17, 95% CI 0.10–0.29, *P* < 0.001) were risk factors for screw loosening after single-level PLIF with CBT screws (Table 3).

**Table 1:** Baseline characteristics and surgical data of patients.

Factors	Total number (*n* = 79)	SL (*n* = 21)	Non-SL (*n* = 58)	*P* value
Demographics				
Age (years)	65.14 ± 9.74	64.62 ± 11.93	62.60 ± 8.87	0.420
Male, *n* (%)	35 (44.3)	11 (52.4)	24 (41.4)	0.385
Height (m)	1.63 ± 0.78	1.66 ± 0.08	1.62 ± 0.07	0.053
Weight (kg)	71.13 ± 13.01	74.29 ± 15.50	69.98 ± 11.94	0.196
BMI	26.83 ± 4.49	26.93 ± 4.17	26.79 ± 4.64	0.905
Follow-up time (mon)	25.38 ± 1.77	25.43 ± 1.43	25.36 ± 1.89	0.884
Surgical data				
Operation time (min)	176.54 ± 41.46	172.62 ± 43.18	177.97 ± 41.12	0.616
EBL (ml)	213.54 ± 74.13	190.00 ± 63.64	222.07 ± 76.29	0.089
Fusion grade, *n* (%)	-	-	-	0.267
I	29 (36.7)	10 (47.6)	19 (32.8)	-
II	42 (53.2)	8 (38.1)	34 (58.6)	-
III	8 (10.1)	3 (14.3)	5 (8.6)	-
ODI (%)				
Preoperative	49.10 ± 6.50	49.52 ± 8.68	48.95 ± 5.60	0.731
6 months	22.52 ± 4.65	23.33 ± 4.53	22.22 ± 4.70	0.353
Final follow-up	21.03 ± 4.50	21.90 ± 4.58	20.71 ± 4.47	0.299

BMI, body mass index; EBL, estimate blood loss; ODI, Oswestry disability index.

**Table 2:** Characteristics of screw-related parameters.

Factors	Total number (*n* = 316)	SL (*n* = 36)	Non-SL (*n* = 280)	*P* value
FS1, *n* (%)	22 (7.0)	9 (25.0)	13 (4.6)	<0.001
Hu	165.18 ± 84.08	179.04 ± 83.63	163.40 ± 83.64	0.294
SA (°)	75.97 ± 7.10	75.72 ± 8.05	76.01 ± 6.99	0.822
CA (°)	10.77 ± 5.72	13.94 ± 6.53	10.36 ± 5.49	<0.001
CBCLs (*n*)	4 (3–4)	2 (2–3)	4 (3–4)	<0.001

FS1, fixed to S1; SA, sagittal angle of the screw; CA, coronal angle of the screw; CBCLs, cortical bone contacted layers.

**Table 3:** Multivariable analysis of radiological parameters.

Variable	OR	95% CI	*P* value
FS1, *n* (%)	3.82	1.12–12.71	0.029
CA (°)	1.07	1.01–1.14	0.039
CBCLs (*n*)	0.17	0.10–0.29	<0.001

FS1, fixed to S1; CA, coronal angle of the screw; CBCLs, cortical bone contacted layers.

**Figure 4:**
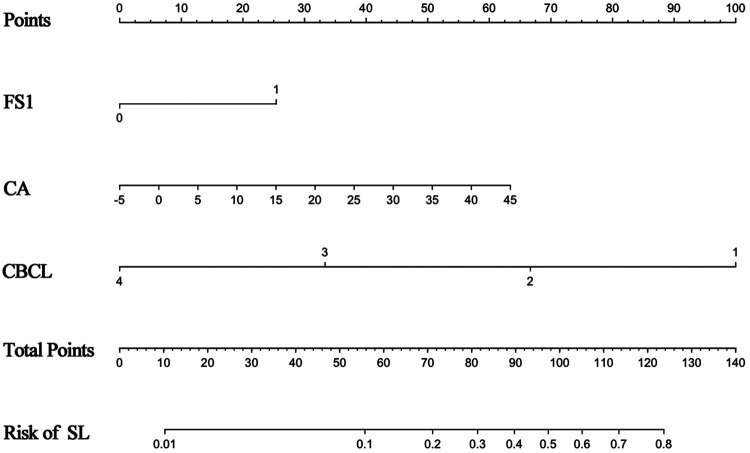
Details of the nomogram.

**Figure 5:**
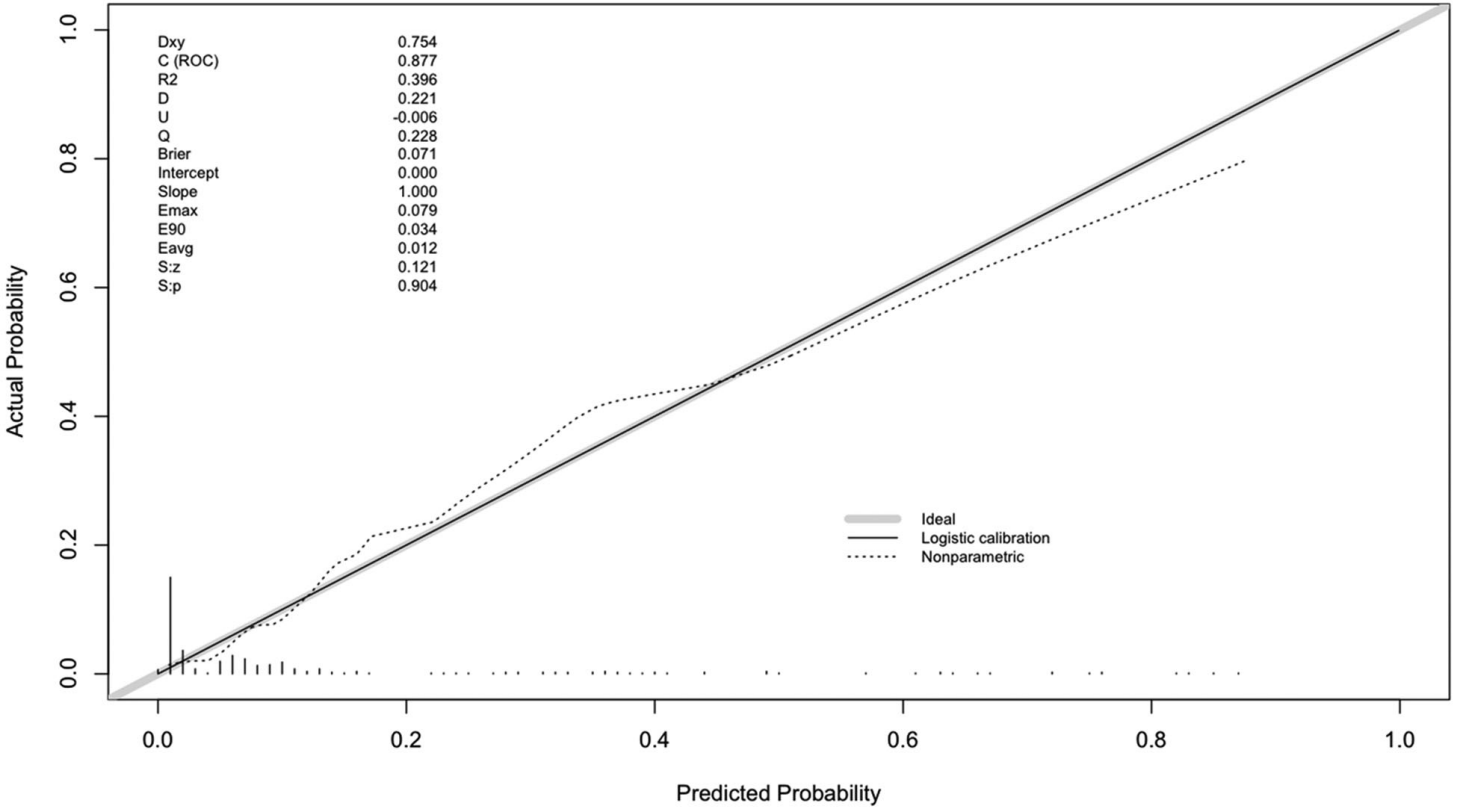
Calibration plot. The calibration of the nomogram was represented by the solid line, and any bias in the nomogram was corrected by the dashed line. The bold gray line indicates the reference line of an ideal nomogram.

**Figure 6:**
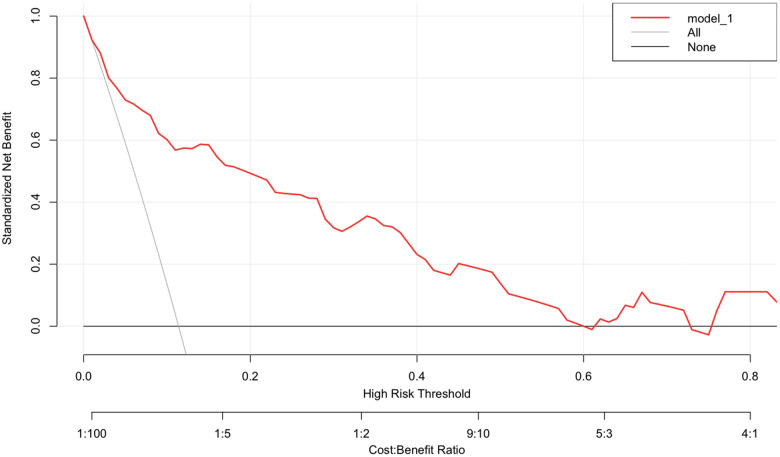
Decision curve analysis of the nomogram. The red line indicates the model. The *x*-axis and *y*-axis display the threshold probability and net benefit, respectively. The gray line represents the net benefit of treating all patients. The horizontal black line displays the net benefit of treating no patients.

The nomogram was conducted by R software (version 4.1.2) with a 0.877 (95% CI 0.818–0.936) C-index, which demonstrated good discrimination and predictive accuracy (Figure 4). Calibration evaluated by the calibration plot of the nomogram was good (Figure 5), and the Hosmer-Lemeshow test was good (*P* = 0.755). The internal validation by bootstraps of 1,000 resamples was excellent, with a 0.880 (95% CI 0.815–0.932) C-index. Decision curve analysis was performed, as shown in Figure 6, and when the threshold probabilities ranged from 0% to 60%, the nomogram showed a positive net benefit, which means clinical interventions implemented in those patients guided by the nomogram could obtain more benefit compared with treating all or treating none.

**Figure 7:**
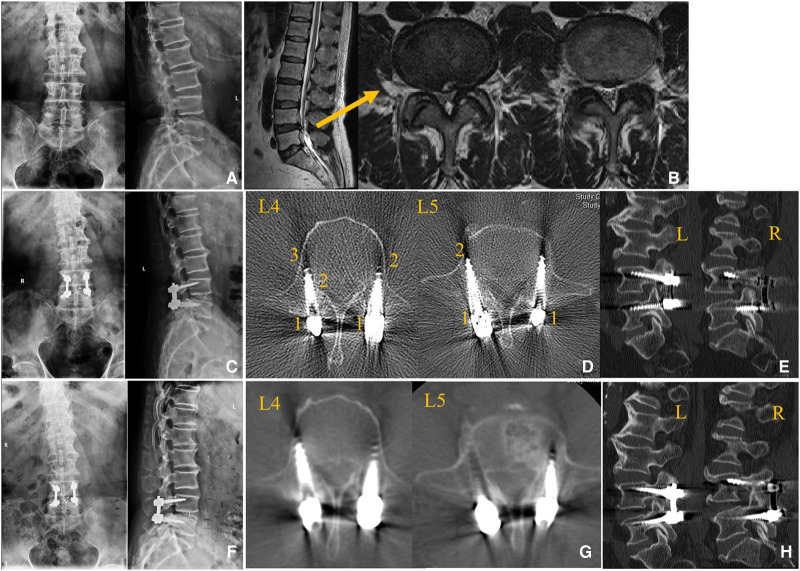
Case of the application of the nomogram. (**A,B**) Preoperative radiological data. (**C–E**) Immediate postoperative radiological data. (**F–H**) Radiological data at 1-year follow-up. (**A**) Lumbar spine x-ray. (**B**) Lumbar spine MRI demonstrating lumbar stenosis at L4/5. (**C**) Postoperative lumbar spine x-ray. (**D**) Postoperative lumbar spine CT scan indicating that the CBCLs of L4L, L4R, L5L, and L5R were 2, 3, 1, and 2, respectively. (**E**) Sagittal view of the lumbar spine CT scan. (**F**) After 1-year follow-up, a lumbar x-ray demonstrated a lucent zone at L4R and L5R. (**G**) Lumbar spine CT scan showing obvious screw loosening at L4L, L5L, and L5R. (**H**) Sagittal view of the lumbar spine CT scan.

Application indication of the nomogram is explained in Figure 7: a 62-year-old male patient underwent single-level PLIF with CBT screws at L4/5. A postoperative CT scan showed that the CA of L4L was 5° and of at L4R was 15°. The CAs of L5L and L5R were 16° and 17°, respectively, and the CBCLs of each screw were 3, 3, 2, and 1. Thus, according to the prediction nomogram, the score of each screw was approximately 45, 59, 92, and 126, which indicated that the incidence of screw loosening was <10%, 11%, 42%, and >80%. At the 1-year follow-up, we identified asymptomatic screw loosening at L4L, L5L, and L5R, which verified the accuracy of the nomogram. Also, the patient maintained asymptomatic at the final follow-up.

**Figure 8:**
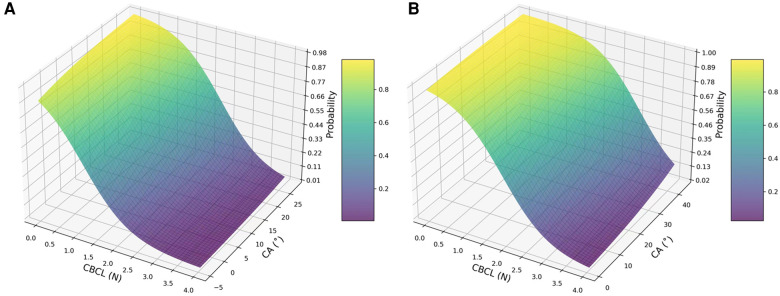
Three-dimensional surface plot demonstrating the impact of CBCL (*x*-axis) and CA (*z*-axis) on the probability of screw loosening after lumbar surgery with the CBT technique. (**A**) Probability of screw loosening when the screw was not instrumented in S1; (**B**) probability of screw loosening when the screw was instrumented in S1.

To indicate whether the screw's location was in S1, three-dimensional surface plots are shown in Figures 8A,B to indicate the impact of CA and CBCLs on the probability of screw loosening.

## Discussion

Screw loosening is common, as reported in PLIF, with an incidence of 1%–60% (7, 10, 13). Risk factors as explored are connected with osteoporosis, incorrect failing loading scenario, insufficient fusion, or screw stress distribution (7); however, most of the research studies have not reached a consensus. CBT screw has comparable pull-out resistance and stability to TPS since it was first proposed in 2009 by Santoni et al. (1). It can provide enhanced screw purchase and preferable interface strength attributed to characteristics of engaging higher density cortical bone even in osteoporosis patients (21–23). Perez-Orribo et al. explored the biomechanics of TPS and CBT and concluded that equivalent stability was found between TPS and CBT fixation (3). Matsukawa et al. found that the screw insertion torque of CBT was 1.71 times higher than that of TPS (24). Thus, theoretically, CBT screw has been proposed to promote pull-out strength and enhance the construct stability. In the present study, we found a 26.6% incidence of screw loosening in 79 samples (11.4% in 316 screws). To investigate the risk factors of screw loosening, we documented and analyzed the mentioned parameters of each screw, which would be more beneficial for surgery, and the results of risk factor analysis showed that three main factors (FS1, CA, and CBCLs) mainly constituted the predict scoring nomogram.

The odds ratio of FS1 was the highest compared to other parameters (OR = 3.82). In our study, there were 22 screws fixed in the S1 vertebra, and 9 of them (40.9%) were found to have an obvious lucent zone in the CT scan. Grigoryan et al. (25) conducted a cadaveric biomechanical study and considered that lumbosacral fixation with CBT screws was stable against loosening, which is contrary compared the results of our study. The reasons of FS1 being concluded as a risk factor of screw loosening were assessed: First, lumbosacral fixation is inherently thought to have a higher risk of screw loosening due to alignment restoration and holding strength (26–28). Second, the learning curve of lumbosacral fixation with CBT is relatively higher. Matsukawa et al. (29) elucidated that the penetrating S1 endplate CBT technique with a mean cephalad angle of 30.7° could provide favorable stability for lumbosacral fixation, while during our work, especially for early cases, it was hard to identify a content position for the instrument in S1 and repeating screw track adjustment might result in instability, and this also occurred in other segments for early cases. Therefore, we considered that experienced surgeons are needed to perform fixation on S1; although the result was not good for FS1, we believe that CBT screw for S1 is an alternative method for fixation due to the reduction of paraspinal dissection and facility for retraction in the sacrum.

With regard to CA and CBCLs played an important role in screw loosening of CBT screw, according to the nomogram. The typical trajectory of the CBT screw contains four parts for the cortical bone to increase the stability of fixation; among these, the lateral par as the starting point is essential. The lateral par is an identifiable structure as an entry point and is less influenced by a degenerative change to provide a good bony reference in the surgery (30, 31). The starting point could also have an influence on CA. Literature works recommended an approximate 10°–14° angle to medial (32, 33), and in our study, the mean CA was 10.36° in the non-SL group and 13.94° in the SL group, which concluded similarly to the previous studies. Matsukawa (4) stated that CA was more variable than SA, and CA might have been derived from differences in the location of the starting point. We believe biochemical studies will be performed to clarify the mechanism in the future.

In the present study, we have documented and provided a reference for the measurement of SA as an angle between the screw line and vertical line because we think that this might reduce the error for measurement of wedge-shaped vertebra in some cases, while some authors recommended a method of the measurement of the angle between the screw and vertebral endplate (33, 34). However, the results showed no statistically significant difference between the two groups, but there was no denial that SA was an important parameter. Zhang et al. (35) conducted a study to compare the fixation failure between PS and CBT and concluded that different failure mechanisms underlay PS and CBT under large vertical displacement, and this may emphasize the characteristic of CBT screws in a sagittal view.

Lower BMD evaluated by dual x-ray absorptiometry (DXA) was significantly associated with screw loosening by influencing the pull-out strength (1, 36, 37); nevertheless, DXA assessed the average value of BMD. In addition to DXA, the use of HU based on a CT scan has been applied and clarified to be a reliable method for BMD evaluation (38–40), which can be used to assess the region involved by each screw. However, literature works revealed that there was no consensus on the HU value to evaluate a low BMD as a risk for osteoporosis. In the current study, BMD around the screw was assessed by the HU value to explore whether BMD would be a risk factor for screw loosening, and the result was negative. This demonstrated that the BMD of the region where screw threaded could not make much difference. Lee et al. (33) reported the HU measurement of cortical bone; however, we have attempted to make the repetition and the results showed poor inter-rater reproducibility due to the thin wall of cortical bone, and we did not adopt the method to replace CBCLs.

The study had some limitations, mainly due to retrospective analysis with small sample size. The surgery with the CBT technique was performed during the learning curve of the early period, and this might contribute to the loosening of the S1 screw. Our study focused on the local view of screws to explore the potential factors of screw loosening; however, we did not include parameters related to fusion because it was hard to judge whether the fusion failure was caused by screw loosening or bone graft. Further studies with experienced surgical techniques will be performed to validate the present study.

## Conclusion

The CBT technique offers an alternative method for lumbar surgery with TPS. Although CBT screws provide good stability for fixation, we have identified significant risk factors for screw loosening. A perioperative evaluation with the nomogram can provide a reliable prediction of screw loosening with CBT screws and contribute to surgical decisions to avoid complications.

## Data Availability

The datasets used and/or analyzed during the current study are not publicly available due to the data being confidential; however, they are available from the corresponding author on a reasonable request.
